# Convergent Variations in the Leaf Traits of Desert Plants

**DOI:** 10.3390/plants9080990

**Published:** 2020-08-04

**Authors:** Muhammad Adnan Akram, Xiaoting Wang, Weigang Hu, Junlan Xiong, Yahui Zhang, Yan Deng, Jinzhi Ran, Jianming Deng

**Affiliations:** State Key Laboratory of Grassland Agro-Ecosystem, School of Life Sciences, Lanzhou University, Lanzhou 730000, Gansu, China; ynmadnan@gmail.com (M.A.A.); wangxt2017@lzu.edu.cn (X.W.); huwg@lzu.edu.cn (W.H.); xiongjl@lzu.edu.cn (J.X.); zhangyh19@lzu.edu.cn (Y.Z.); yvette456ding@163.com (Y.D.)

**Keywords:** convergence, leaf traits, desert plants, phylogenetic signals

## Abstract

Convergence is commonly caused by environmental filtering, severe climatic conditions and local disturbance. The basic aim of the present study was to understand the pattern of leaf traits across diverse desert plant species in a common garden, in addition to determining the effect of plant life forms (PLF), such as herb, shrub and subshrub, phylogeny and soil properties on leaf traits. Six leaf traits, namely carbon (C), nitrogen (N), phosphorus (P), potassium (K), δ^13^C and leaf water potential (LWP) of 37 dominant desert plant species were investigated and analyzed. The C, N, K and δ^13^C concentrations in leaves of shrubs were found higher than herbs and subshrubs; however, P and LWP levels were higher in the leaves of subshrubs following herbs and shrubs. Moreover, leaf C showed a significant positive correlation with N and a negative correlation with δ^13^C. Leaf N exhibited a positive correlation with P. The relationship between soil and plant macro-elements was found generally insignificant but soil C and N exhibited a significant positive correlation with leaf P. Taxonomy showed a stronger effect on leaf C, N, P and δ^13^C than soil properties, explaining >50% of the total variability. C_3_ plants showed higher leaf C, N, P, K and LWP concentration than C_4_ plants, whereas C_4_ plants had higher δ^13^C than C_3_ plants. Legumes exhibited higher leaf C, N, K and LWP than nonlegumes, while nonlegumes had higher P and δ^13^C concentration than legumes. In all the species, significant phylogenetic signals (PS) were detected for C and N and nonsignificant PS for the rest of the leaf traits. In addition, these phylogenetic signals were found lower (*K*-value < 1), and the maximum *K*-value was noted for C (*K* = 0.35). The plants of common garden evolved and adapted themselves for their survival in the arid environment and showed convergent variations in their leaf traits. However, these variations were not phylogenetics-specific. Furthermore, marks of convergence found in leaf traits of the study area were most likely due to the environmental factors.

## 1. Introduction

Convergence is the tendency or ability of different organisms to evolve phenotypically related traits under similar natural environments and it commonly appears in extreme ecological conditions [1]. Similar environments, microsites and living behaviors should cause similar pressure for selectivity that consecutively supports morphophysiological plant traits, which exploit the competitive ability and fitness under such environments and bring about evolutionary convergence among species despite many differences among their forefathers [2,3]. Probably, that is the reason many plants show convergence, e.g., alpine plants have thick small leaves and cushion form of growth in a significant number of lineages [2] and many annuals succulents plants having small, bright or prickly photosynthetic surfaces dominate a number of desert areas [2,4].

Environmental filtering decides which species can survive in their populations in a specific environment [5]. This deterministic filtering finds the existence probability of species and low existence probability species are considered as sifted out [6]. Abiotic conditions influence the existence probability of species as well as their abundance. These two effects on existence and abundance of species are the marks of trait convergence [7]. Habitat filtering is believed to be one of the main divers for community assembly, which causes trait convergence in severe environmental conditions [8].

Competitive ability and fitness of the organisms depend on environmental conditions [9]. Mostly, close members are strong competitors for each other because of their phenotypic resemblance [10]. The different plant traits and growth forms that are not suitable in a specific ecological condition (e.g., *Columnea glabra* in tropical rain forests) will be supportive for adaptations under different environmental situations (e.g., *Silene acaulis* and *Diapensia lapponica* cushion shrubs with thick small leaves in arctic tundra and alpine). Different plants have different levels of adaptation that lead to convergence and are considered as one of the main drivers of diversification. Physical situations can limit the survival and development of many plants because of harsh environmental conditions, at which point convergence is marked. The species start sorting in favor of local environmental conditions and convergence among different species may start along environmental gradients [2].

Based on the convergence theory of the unrelated species (taxonomically) [11,12], leaf traits are commonly considered in the ecology to clarify the links among species characteristics, division, habitats and their role in the community assembly [13,14]. A consistent trend about interspecific variations in the plant leaf traits has been found in previous studies across different environments at regional and global scales [15,16]. This trend strengthens the plant strategy theory through expounding arrangements in plant traits for establishment in different environments [17,18].

Leaf attributes and environmental relationship studies have illustrated strong tradeoffs in leaf trait variation over different biomes [15,19]. A strong relationship among different traits of the leaf, such as leaf mass area (LMA) and life span (LLS), is considered as a variation spectrum among species that shows leaf structural tradeoff globally. Whether these patterns are in a specific or single biome is less explored, but leaf survival techniques are probably going to be found on a limited scale of explicit ecological stressors [20,21].

The functional traits of the plant are studied progressively as a basic framework to comprehend species response towards ecological changes and differences in their distribution [22,23]. They deliver a better understanding of concurrent changes in desert areas, nutrient accessibility that differentially affects co-occurring desert plant species and convergent evolution [24,25]. It has been found that these traits strongly correlate with soil moisture contents [26] and also play a key role in finding competitive hierarchies among different plant species [23].

Leaf traits of various plant taxonomic groups across different biomes have significant ecological and evolutionary values. Thus, without knowing phylogeny, it is impossible to check the adaptive importance of leaf traits [27]. The different trait combinations in plant species result from tradeoffs [28]. Plant traits with similar genetic background and evolutionary history as a result of convergence are difficult to elaborate [29]. However, many studies and models have emerged recently to clarify the phenomena and factors behind the mechanism of convergence of leaf traits. However, the possible potential mechanism behind convergence and the influence of phylogeny on leaf traits is still unclear and is a considerable challenge [29]. Moreover, the degree to which interaction between plant functional traits and different environmental gradients pertains to specific local and regional situations needs further exploration [12].

The Guazhou common garden (GCG) was established in 1986 at the provincial level with the approval of Gansu’s Provincial Government, China and it earned national status in 1992. It is located in Guazhou County (on the ancient Silk Road) and at the junction of temperate deserts, extremely arid deserts and typical deserts in Central Asia. It is the only national-level reserve that protects extremely arid desert ecosystems and their biodiversity in China [30,31]. The majority of the plant species of GCG (>60%) are native to Central Asia [30]. The species grown in the GCG have faced different environmental conditions and stresses since the GCG was established.

Thus, the present study was designed to understand the changing trends of leaf traits across diverse desert plant species/taxon in the common garden. Three key assumptions were constructed: (i) Leaf morphological and physiological traits converge on a similar pattern among plant species grown under common garden for a long time ago; (ii) The convergence pattern is phylogenetics-specific across different plant species; and (iii) To document the patterns of different leaf trait for all 37 plant species and different life forms. For this purpose, the study was focused on the leaf traits (C, N, P, K, δ^13^C and LWP) of 37 desert plant species that were collected from GCG (Table 1).

## 2. Results

### 2.1. Species Composition, Leaf Traits and Soil Parameters

**Figure 1:**
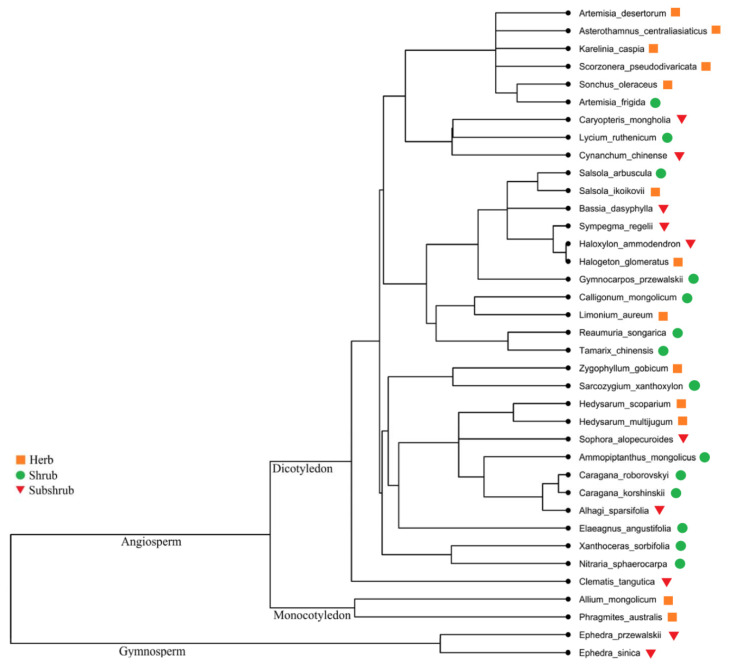
Phylogenetic structure of 37 species in the study area.

A total of 37 desert plants was collected belonging to 18 families and 32 genera; among all 37 species, Fabaceae (7), Asteraceae (6), Amaranthaceae (6), Ephedraceae, Tamaricaceae and Zygophyllaceae (3 each) and so on (Table 1). For plants, different functional groups the species were categorized by different parameters: (i) herbs (13), shrubs (14) and subshrubs (10), (ii) monocots (2) and dicots (33), (iii) C_3_ species (32) and C_4_ species (5), (iv) gymnosperms (2) and angiosperms (35), (v) annual (4) and perennial (33), (vi) legumes (7) and nonlegumes (30). The chief plant habit was shrubs (Table 1; Figure 1).

In general, for all 37 plant species, leaf C values ranged from 262.25 to 772.99 with the mean value 463.97 mg g^−1^; leaf N values ranging from 12.99 to 53.40 with mean value 25.27 mg g^−1^; leaf *p*-values 0.42–2.51 with the mean value 0.85 mg g^−1^; leaf K values 4.13–26.35 with mean value 11.60 mg g^−1^; leaf δ^13^C values −28.98 to −13.62 with the mean value −24.66 and leaf LWP values ranged from −19.60 to −3.01 with the mean value −7.68 MPa (Table 2). In the study area, the soil was alkaline with its pH 8.97; while soil organic C(SOC), soil total N(STN) and soil total P(STP) concentrations were 2.58, 0.239 and 0.343 mg g^−1^, respectively (Table 3).

### 2.2. Patterns of Leaf Traits among Different Functional Groups

**Figure 2:**
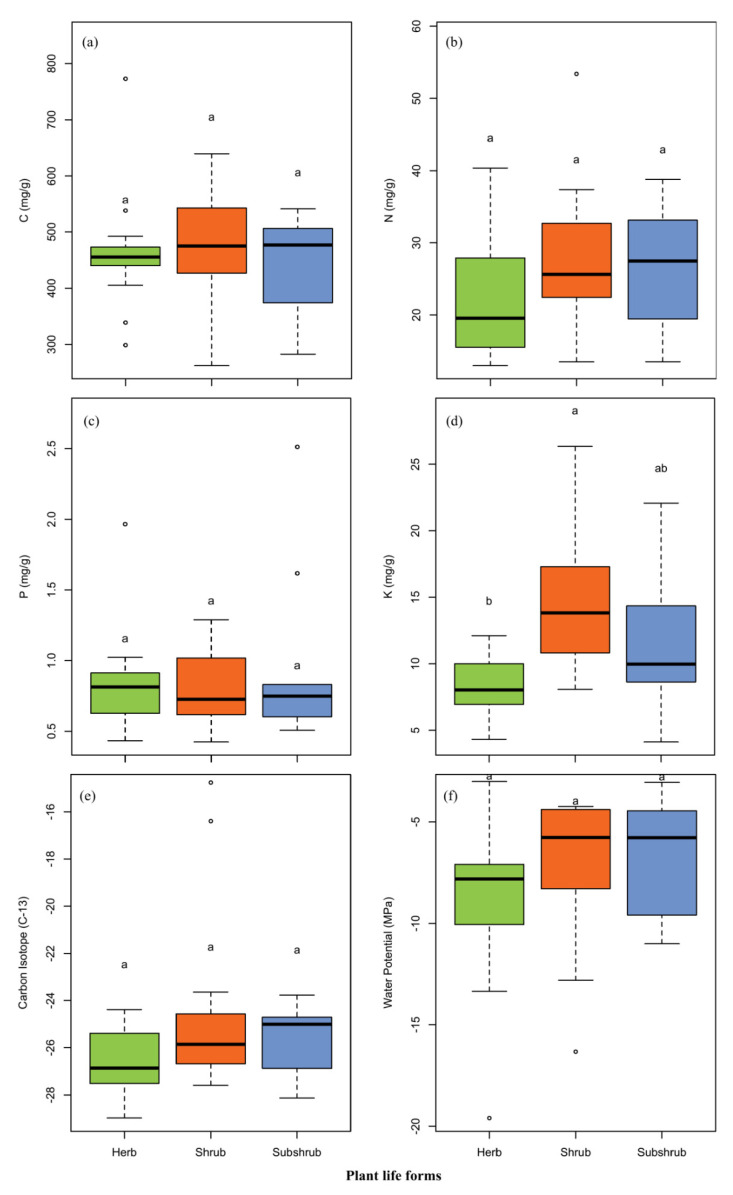
Leaf traits data for species in different life forms (such as herb, shrub, subshrub): (**a**) leaf carbon concentration (C); (**b**) leaf nitrogen concentration (N); (**c**) leaf phosphorus concentration (P); (**d**) leaf potassium concentration (K); (**e**) carbon isotope C^13^ concentration (δ^13^C) and; (**f**) leaf water potential (Ψ_l_). Data presented beyond whiskers represent outliers and letters indicate significant level (*p* < 0.05) among life forms (Tukey’s HSD test).

Element concentrations of six leaf elements were analyzed for different plant life forms. The mean C, N and K concentrations and δ^13^C in leaves of the shrub were found greater than the herbs, and subshrubs, respectively (Table 2; Figure 2). The mean P and LWP concentrations in the leaves of subshrub were found higher than the herb and shrub, respectively (Table 2; Figure 2). ANOVA results analyzed the effect of life form on different traits (Table 2). No significant differences in concentrations of C, N and P, as well as δ^13^C and LWP were found. Whereas, in the case of K, there were significant differences observed among the different life forms.

The C^3^ plants had higher leaf C (significantly), N (significantly), P, K and LWP concentration than the C_4_ plants; while, C_4_ plants had significantly higher δ^13^C concentration than C_3_ plants. ANOVA results for C_3_ and C_4_ plants showed that leaf C, N and δ^13^C were significantly different; whereas no significant differences in the concentrations of P, K and LWP (Table 4). Legumes exhibited higher leaf C, N, K and LWP than the nonlegumes; nonlegumes had higher P and δ^13^C concentration than legumes. ANOVA results for legumes and nonlegumes showed no significant differences in C, N, P and K concentrations, δ^13^C, and LWP (Table 4).

### 2.3. Correlations between Different Leaf Traits

**Figure 3:**
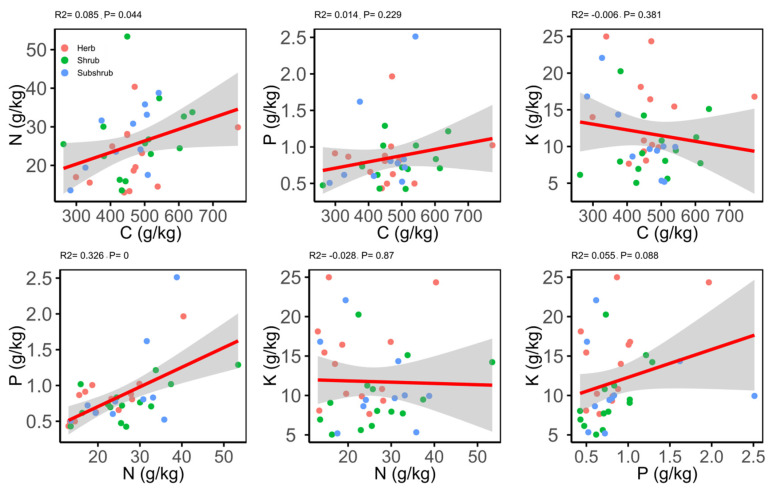
Relationship between different chemical traits of the leaf.

Both Pearson’s and phylogenetic independent cross (PIC) correlation matrix illustrated different correlations for the leaf traits (Table 5). The results of the Pearson’s correlations showed that C was significantly positively correlated with N and negatively correlated with δ^13^C; whereas, no correlation was found with P, K and LWP. The N exhibited a positive correlation with P and no correlation for the rest of the traits (Figure 3). On the other hand, K and LWP showed no correlation with C, N, P and δ^13^C (Table 5).

However, the significant correlation between leaf C and δ^13^C disappeared after the use of PIC method, while the significant correlation between N and δ^13^C; P and K, δ^13^C, LWP; and K and LWP was found (Table 5). Moreover, in all the species significant phylogenetic signals (PS) for C and N were detected and nonsignificant PS for the rest of leaf traits (Table S1). However, the low phylogenetic signals (*K*-value < 1) were found for all the leaf traits and the maximum *K*-value was noted for C (*K* = 0.35).

**Figure 4:**
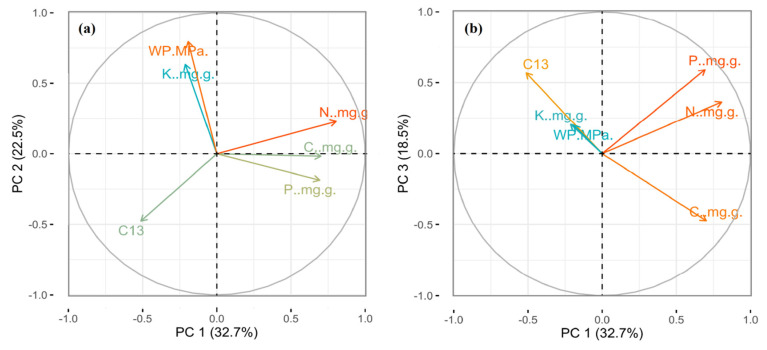
The expression of six leaf traits on the three principal components analysis (PCA) axes at the species level (*N = 37*). (**a**,**b**) Loading values of six leaf traits for PC axis 1, 2 and PC axis 1, 3, respectively. Different colors show the contribution of each variable.

The factor loading of the first three axes was considered for six leaf traits by using principal components analysis (PCA) (Table S2; Figure 4). On the first PCA axis was mainly N, C and δ^13^C loaded; the second axis was mainly loaded by P and K; while, LWP loaded on the third axis, and the explained variance of each axis was 32.7%, 22.5% and 18.5% of the total variability, respectively (Table S2).

### 2.4. Relationships between Traits of Plant and Soil

**Figure 5:**
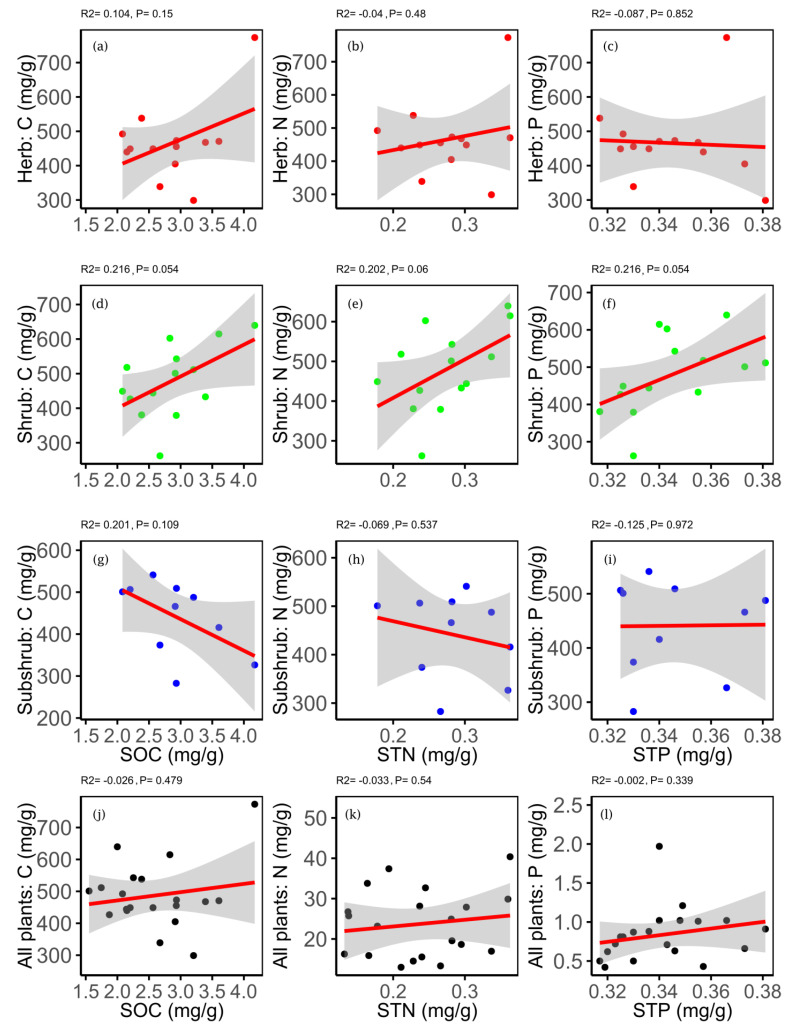
Relationship between leaf carbon (C) and soil organic carbon (SOC) (in panel **a**,**d**,**g**); leaf nitrogen (N) and soil total nitrogen (STN) (in panel **b**,**e**,**h**); and leaf phosphorus (P) and soil total phosphorus (STP) (in panel **c**,**f**,**i**). Red dots used for herb (in panel **a**,**b**,**c**), green for shrub (in panel **d**,**e**,**f**), blue for subshrub (in panel **g**,**h**,**i**) and black for all plants (in panel **j**,**k**,**l**).

The correlation between soil and plant macro-elements was generally insignificant, but soil C and N were found significantly positively correlated with the leaf P (Table S3). For PLF, SOC and STP were significantly correlated with the leaf C and N, whereas STN showed a marginal significant correlation with leaf N of shrubs (Figure 5).

**Figure 6:**
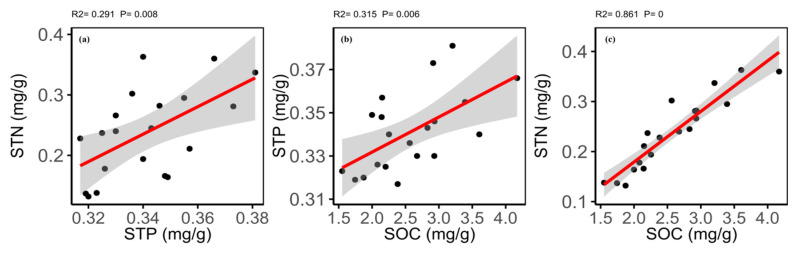
Relationship between (**a**) soil total phosphorus (STP) and soil total nitrogen (STN); (**b**) soil organic carbon (SOC) and soil total phosphorus (STP); and (**c**) soil organic carbon (SOC) and soil total nitrogen (STN).

The correlation matrix showed different relationships for soil elements and soil variables (Table S4). The results of Pearson’s correlations showed that the SOC was significantly positively correlated with the STN and STP. The STN was significantly positively correlated with the STP (Figure 6). Soil pH showed no correlation generally for the soil elements and variables except a negatively significant relationship with soil moisture contents (SMC) and soil electro-conductance; while, the SMC showed a significantly positive correlation with the SOC and STN (Table S4).

### 2.5. Taxonomic Effects on Leaf Traits at Species and Family Level

The six analyzed leaf traits varied considerably across the species (Figure S1; Table S5). At the species level, more C was accrued in the leaves of *Artemisia desertorum* and *Artemisia frigida*; more N in *Nitraria sphaerocarpa* and *Allium mongolicum*; more P in *Caryopteris mongholia* and *Allium mongolicum*; more K in *Ammopiptanthus mongolicus* and *Reaumuria songarica*; more δ^13^C in *Salsola ikoikovii* and *Haloxylon ammodendron* and more LWP in *Hedysarum scoparium* and *Sympegma regelii*.

The six analyzed leaf traits also varied considerably across all 18 families (Figure S2; Table S6). At the family level, more C was accrued in the leaves of Sapindaceae and Asteraceae; more N in Nitrariaceae and Amaryllidaceae; more P in Lamiaceae and Amaryllidaceae; more K in Tamaricaceae and Amaranthaceae; more δ^13^C in Polygonaceae and Amaranthaceae and more LWP in Solanaceae and Caryophyllaceae.

### 2.6. Partitioning of Variance in Leaf Traits

The general linear models (GLMs) were used to access the roles of taxonomy and soil properties on the leaf traits. Full models have explained a considerable part of variances in the leaf traits (Table 6). The model assumed for 31–95% of the total variability. Taxonomy explained 31–82% variance and soil properties explained 0.3–18% variance of the total variability in traits of the leaf. Moreover, these two factors (taxonomy and soil properties) had strong varied explanatory powers, as the taxonomy showed stronger effects on the leaf C, N, P and δ^13^C, while soil properties also explained stronger effects for the variances in the same leaf traits (Table 6).

## 3. Discussion

The present study here provides new insight into the relationships between different leaf traits and their phylogenetic patterns of desert plants.

### 3.1. Patterns of Leaf Traits in All Species

The pattern of leaf traits concentration for all 37 species was investigated (Table 2). In the present study, it is noted that the mean leaf C concentration of all 37 plant species (Table 2) was higher than that in leaves of plant species studied across the China [32], plants of the Taklamakan Desert [33] and especially higher than herbs of the Yucatan Mexico [34]. Results of the current study further investigated that the mean leaf N concentration (Table 2) significantly higher than the global plants [35], China’s terrestrial ecosystems [32], herbs studied across China [36] and markedly lower than the KMUNR desert plants [37]. However, mean leaf P concentration (Table 2) was considerably lower than that of China’s terrestrial ecosystems [32], global plants and KMUNR desert plants [35,37], plants of temperate Alxa Desert [38] and global plants [39], which were attributed the low P availability in the desert soils [40]. Plants likely allocate nutrients to leaves first to ensure and secure their growth [41]. Leaf nutrient concentrations play a basic role in the plant ecophysiology and ecosystem functioning [42]. Plant growth is the main process controlling C input in the terrestrial ecosystems, which requires partially 16 elements in different amounts [43]. These elements are closely coupled with the C-sequestration processes [32,43]. In ecosystems, C, N and P are considered major limiting nutrients for C-sequestration [44]. The C is a basic element that constitutes the plant structure; N is an important element for enzymes; and P for nucleic acids and membrane lipids [32]. Plant species with low or imbalanced N and P availability may find it is difficult to obtain sufficient amounts of C, N and P for different physiological functions and their survival [45]. The P is crucial for plant growth and to maintain WUE, particularly in arid environments [46]. Moreover, the photosynthetic rates are associated to leaf nitrogen (N) and phosphorus (P) concentrations [42] and with carbon, they are considered as the premise of ecological stoichiometry [47] and basic elements of elementome and biogeochemical niche [45]. It is helpful to evaluate the ecological traits from the elemental formation of living beings [42,48]. Consequently, the Guazhou plants showed higher leaf N concentration but lower leaf P concentration as compared to previous studies. Desert plants need to allocate large amounts of the P-rich RNA to meet higher growth rates [49], and also prompt and certain need of higher N content to support and enhance enzyme resistance for the survival in extremely arid conditions [50]. The P is also essential to maintain WUE and growth, especially in the arid environments [46]. Thus, comparing with P, desert plants have a higher allocation of N. Moreover, the desert soils had substantially lower solubilization than the grassland [51].

Potassium is one of the major elements along with the N and P, which is necessary for many physiological processes and plant growth [52]. More than 60 enzymes are activated by the potassium that plays a vital role in the regulation of osmosis and stomata, as well as transpiration [53]. The adverse effects of drought on the concentration of P and K in the desert ecosystems may cause additional indirect harmful effects on the fitness of plants [54]. Mean leaf K (Table 2) was found lower than that of herbs studied across China [55] and plants of southwestern China [56], which is due to low K availability in the desert soils [57]. In this study, the leaf δ^13^C mean value (Table 2) was comparatively larger than the plants of eastern Mount Tianshan [58]. As in plants, δ^13^C based on the ratio of intercellular: ambient CO_2_ concentration (ci/ca) that shows a balance of inward CO_2_ diffusion, stomatal conductance (g) rate, and CO_2_ assimilation (A) rate [58] and it is considered as a sensitive indelible sign of physiological changes [59]. The δ^13^C increases with decreasing atmospheric pressure. In arid environments, the water availability negatively affects the concentration of δ^13^C [60], and more water availability boosts stomatal conductance that leads CO_2_ to internal leaf and resultantly the ci/ca ratio increasing and finally the δ^13^C concentration decreasing [58]. The regulation of the LWP is attributed as a crucial process for plants and it is optimally regulated by plants on the basis of described tradeoff. The LWP plays an important role in stomatal conductance, CO_2_ uptake, xylem functioning, water supply and the growth of cells. When plants uptake CO_2_, then they drop their LWP through transpiration to avoid a reduction in growth [61]. However, the LWP mean value (Table 2) was found significantly lower than all plant types of the USA [62]. In arid environments, low LWP is common; whereas, in these environments, water use efficiency can be improved by reducing H_2_O and CO_2_ balance. Low LWP can actuate adverse impacts on the assimilation of CO_2_ and WUE by damaging photosynthesis and decreasing mesophyll conductance [63] that ultimately affects plant growth.

### 3.2. Patterns of Leaf Traits among Different Functional Groups

For plant life forms, desert shrubs accrued more C, N and K and showed more LWP than the herbs and subshrubs; and subshrubs showed higher concentration of P than the herbs and shrubs; while, the value of δ^13^C was found roughly similar for all life forms, such as herbs, shrubs and subshrubs (Table 2). Leaves C, N and K are tightly coupled with main biological functions, like photosynthesis, respiration and water use [38,64]; while, leaf water potential depict the water status of the plant [65], the key process for ecosystem functioning [61], influenced plant productivity, photosynthesis and growth [66]; so, desert plants regulate optimal leaf water status for their survival which is a basic constituent of plant functioning [61]. This is the reason shrubs dominate most of the desert lands [67]. In the present study, mean leaf C and N concentration of shrubs (Table 2) were considerably higher than that in leaves of terrestrial plant species studied across China [32]. The P concentration of shrubs (Table 2) was found lower than that in leaves of herbs studied across China [36] and China’s terrestrial ecosystems [32], which is due to low P availability in desert soils [40]. Moreover, in shrub’s higher N and lower P concentrations than herbs [38] and subshrubs (but P concentration was lower than subshrubs); rather supporting the idea that fast-growing plants (short-lived) have higher N and P concentrations than slow-growing plants (long-lived) [38,68]. However, the mean leaf K of shrubs (Table 2) was found higher than plants of temperate Alxa Desert [38], herbs studied across China [55] and lower than the plants of southwestern China [56]. The mean leaf δ^13^C (Table 2) was found roughly similar for all life forms (such as herbs, shrubs and subshrubs, respectively), which may be due to soil moisture in arid environments [58]. LWP mean value (Table 2) was found significantly lower than all plant types of the USA [62]. Low LWP is common in arid areas [61] and in these areas, WUE can be enhanced by reducing H_2_O and CO_2_ balance [63].

The C_3_ plants have higher leaves C, N, P, K and LWP than the C_4_ plants while; C_4_ plants have significantly higher δ^13^C concentration than C_3_ plants (Table 4). The C_3_ plants have higher N and P concentrations than the C_4_ plants [37,69]. Plants showed a high growth rate (HGR) usually contains rich P concentration and P-rich RNA to meet the energy requirement of different metabolic processes [49]. Low leaf N and high WUE of a plant specified the high values of δ^13^C [70]. Thereby, C_4_ plants have higher values of δ^13^C and low leaf N and P than C_3_ plants (Table 4) [37]. The C_4_ plants generally have more C and K concentrations than C_3_plants because of their high photosynthetic rate and WUE [38]. Conversely, we noted that the C_4_ plants have lower leaves C, K and LWP than C_3_ plants (Table 4), which suggests the divergent adaptations of C_4_ plants; however, low LWP in C_4_ plants linked to photosynthesis decline in the C_4_ plants [71].

Legumes have higher leaves C, N, K and LWP than nonlegumes, and nonlegumes have higher P and δ^13^C levels than legumes (Table 4). Legumes accumulated more C and N than nonlegumes [72]; because they can exchange C for N with N_2_-fixing symbionts [73] and also use C as fuel for N_2_-fixation [74], so the legumes have high C. While, higher leaf N in legumes depicting their higher WUE and photosynthetic capacity [75]. Generally, legumes are P-rich plants and used this P for nodule formation, plant biomass and different physiological functions [76]. Conversely, it is noted in the present study that legumes have lower P than nonlegumes (Table 4), which indicates the low P contents in the desert soils [40]. P deficiency directly affects the nodulation and plants show smaller nodules than normal ones [77]. The present study determined that legumes have higher K than nonlegumes, which is required for activation of different enzymes in legumes, including the nitrogenase [77]. Moreover, it is found that nonlegumes have higher values of δ^13^C, low leaf N and LWP than legumes (Table 4), which suggests that nonlegumes have high WUE [70].

### 3.3. Correlations between Different Leaf Traits

All the leaf traits showed a positive or negative correlation with each other using the PIC method, suggesting that leaf traits are phylogenetically conserved. Leaf C, N and P exhibited a significant positive correlation with one another either with or without considering their phylogeny (Table 5), demonstrating that these leaf traits share correlated evolutionary changes [19]. The relationships between different leaf traits can be related to their chemical properties and biochemical function [19,78]. In all species, significant phylogenetic signals (PS) for C and N were detected and nonsignificant PS for the rest of leaf traits. It is inferred that phylogeny strongly influenced C and N thus these traits may be phylogenetically more conserved. Low phylogenetic signal (*K*-value < 1) was found for all leaf traits (Table S1), indicating that leaf traits were mainly influenced by climatic factors [79].

It was observed in the present study, that the first PCA axis was mainly loaded by C, N and δ^13^C; the second axis by P and K; while, as well as LWP loaded on the third axis and explained variation of each axis was found 32.7%, 22.5% and 18.5% of the total variability, respectively (Table S2; Figure 4). The elements of the first PCA axis were primarily necessary for plant structure, photosynthesis and protein synthesis [19,32]. The elements associated with the second and third PCA axis were essential for enzyme activity, stomatal conductance, regulation of transpiration and plant water supply [53,61].

### 3.4. Relationships between the Different Traits of Plant and Soil

SOC, STN and STP are significant markers of soil fertility and productivity [80]. SOC directly affects the ecosystem’s production capacity and shows the response of an ecosystem to the environment [81]. STN and STP are basic elements for plant growth that directly affect the photosynthesis and different processes related to productivity [82]. The availability of soil nutrients is considered one of the major influential factors that affect leaf element concentration. As globally, plant K concentration is greatly affected by the availability of soil K [19]. Previous studies showed that soil nutrients affect stoichiometric ratios of plant nutrients; as the leaf C, N and P contents were found positively related to soil C, N and P contents [83]. It was observed that SOC and STN positively correlated with leaf P (Table S3), signifying that when the SOC and STN level increased in soil then may increase C and N uptake level in plants; and after all plants absorb more P due to elemental homeostasis [84]. Thus, SOC and STN were the main factors in the present study that affected the plant macro-elements stoichiometry because SOC and STN were also positively evidently correlated to STP (Table S4). Moreover, the result is corroborated by earlier studies, showing that STP is noticeably linked with SOC and STN concentration [85] and scarcity of STN directly affect P concentration in plants [86]. Therefore, the SMC showed a positive significant relationship with the SOC and STN, ensuing in variations in leaf P contents due to interactive relationships among SOC, STN and STP. The results of the present study corroborated the findings of a recent study [33] that the source of plant nutrients was not merely soil but also groundwater/SMC. Furthermore, soil pH and SMC also affect concentration and storage of SOC, STN, and STP [81,87]. However, soil pH showed a negative significant relationship with SMC [88] and SEC [89] in this study (Table S4), suggesting that soil pH may directly affect the SMC and salts solubility, but indirectly affect the SEC. While the SMC also plays a vital role in SEC and SEC directly related to salinity (presence of soluble salt in the soil). The negative relationship between soil pH and SEC is not linear but in the form of a power function, because soil texture, soil minerals, soil temperature and soil moisture also affect the SEC [89].

### 3.5. Taxonomic Effect on Leaf Traits

The results showed that leaf element concentrations varied significantly across all the species (Table S5; Figure S1) and families (Table S6; Figure S2). Taxonomic variance explained up to 82% of the variation (Table 6). Though, the degree of variance components owing to taxonomy varied extensively between leaf elements. The taxonomic role of leaf C, N, P, K, δ^13^C and LWP variation was found considerably higher than the role of soil properties. Recent studies have affirmed that taxonomic affiliations [19,68] and plant phylogeny greatly affects the concentration of plant nutrients [37,38], their uptake and mineral concentrations in various plant parts as well as in the leaf [32].

Some plants can uptake/accrue certain elements in a huge amount [19]. For example, gymnosperms can accumulate more C than angiosperms [90]. The accumulation of C is influenced by plant functional type. Unlike gymnosperms, angiosperms have a lack of highly lignified and woody stems but have nonstructural carbohydrates in high quantity, showing how the phylogenetic differences affect the C concentrations [91]. It was found that the C_3_ plants can accumulate more K than the C_4_ plants [38]. Consequently, variation in concentrations of leaf traits among different life forms, taxonomic groups or functional groups is probably related to variations in the structure of leaf tissues or their osmotic fractions, and due to selective uptake of different nutrients by plant species [19]. Moreover, variation in concentrations of leaf traits is also influenced by vegetation type, climate, geography and availability of soil nutrients [32].

### 3.6. Partitioning of Variance in Different Leaf Traits

In the GLM results of the present study, taxonomy and soil properties (two factors) collectively accounted for >50% of the variations in leaf element concentrations, except K and LWP, explained >30% of the variation. Though, the soil properties had shown very low explanatory power than taxonomy, only explaining 0.3–18.4% of the variation (Table 6). In the study area, the mean annual temperature (MAT) and mean annual precipitation (MAP) were 9 °C and 45 mm, respectively; whereas, AI was <0.02 that signifies that it is a hyperarid region [27,31]. Precipitation and temperature can directly affect the concentration of plant elements by changing the nutritional distribution between organs and the concentration of metabolic-related elements, or indirectly affecting the N or P concentration of leaves by changing soil vegetation composition and biogeographic processes [92]. Desert plants show adaptive variations in such types of extreme arid environments [37]; as well as adaptations and evolution for the long term in desert plants make them able to develop some special structures and strategies for their survival [93]. For example, plant species with rich contents of leaf N and P generally grow faster [94], and hence changes in the concentrations of leaf traits can be related to the physiological demand of plant species [19]. However, soil moisture and temperature also can affect the uptake of N and its utilization by plants [95]. For example, higher macronutrient concentrations in the leaves are adaptive characteristics of plants that boost the metabolic processes in response to the environmental stress [19]. The variations in leaf element concentrations are largely influenced by taxonomy [38]. Moreover, the taxonomy and soil properties explained most of the variance in leaf C, N, P and δ^13^C (Table 6). The concentrations of N and P regulate the productivity and sequestration of C in the terrestrial ecosystems [32]. Whereas, the availability of these elements in leaves depends largely on soil water contents [96]. Infrequent precipitations limit the soil weathering process, mineralization and finally lead to the slow release of P from primary material [97]. As well as, the availability of P is also limited by precipitation, the ability of phosphorus solubility with other elements and adsorption [98]. All plants need 17 elements (some plants need additional four elements) for the completion of their life cycles, and plants obtain C, H and O from air and water; while remaining 14 elements from the soil [99]. Some of them are not directly available (e.g., K, Zn, Fe, etc.), especially in the arid and calcareous soils for desert plants [38].

## 4. Materials and Methods

### 4.1. Site Description

The study site was selected as a common garden, which is located in Guazhou County of Jiuquan city (desert control station) on the northwest of Gansu Province, China (40°31′ N, 95°46′ E and elevation 1179 m above sea level). The mean annual temperature (MAT) is 9 °C, mean annual precipitation (MAP) is 45 mm and annual evaporation is about 3000 mm [27]. The Shule River runs in this region and causes salinity due to high surface evaporation. The aridity index is <0.02 that signifies a hyperarid region [27,31].

### 4.2. Data Collection and Methods

Data were collected during August–September 2018. The mature, fully expanded and sun-exposed leaves or leafy shoots were gathered from 3 to 5 healthy plants of every species. The total fresh mass of leaves was collected (more than 100 g) for every plant species, which were placed in paper bags (one bag for one plant species was used) after the uniform mixing for subsequent laboratory analysis. The stored leaf samples (in paper bags) were oven-dried at 80 °C for about 24 h to a constant weight. The dried plant material was ground (each plant sample was ground separately) into a fine powder by using the high-speed ball mill grinder (MM200, Retsch, Haan, Germany) for the chemical analysis. The leaf samples of 37 plant species (abundant species) were collected and investigated (Table 1). The plant species were divided into three functional groups concerning their life forms/habit (such as 13 herb, 14 shrubs, 10 subshrubs) by following the descriptions reported in Halophytes in China [100].

Soil samples were collected from 20 different places of the study site (GCG) at the depth of 0–20 cm in triplicate by using soil auger, where every soil sample replicate (>200 g) comprised a mixture of three soil cores. Fresh soil samples were placed in small boxes (aluminum) and weighed (in situ) by using an electronic balance. After sampling, all soil samples were brought to the laboratory and dried at 105 °C for about 24 h to determine SWC. Then, air-dried soil samples were sieved (2 mm), and total C (in the leaf and soil samples) was measured by the volumetric method (ferrous sulfate titration after oxidation of potassium dichromate). Soil total N and plant N was determined by the Automatic Kjeldahl Analyzer following the Kjeldahl method. Ammonium (NH_4_^+^-N) and nitrate (NO_3_—N) were obtained by using a TOC-TN analyzer. The total P (in the leaf and soil samples) was determined by the ammonium molybdate method [101] and K concentrations (in the leaves) by using a flame photometer (FSP6650). The soil electrical conductivity [102] and soil pH were determined using a soil: water ratios of 1:5 and 1:2.5, with an EC meter (DDSJ-318) and pH meter (Sartorius PB-10), respectively. Predawn leaf water potential was measured in the field (GCG) by using a potentiometer (LWP4C). To compute carbon isotope (δ^13^C), dried leaf samples were ground in the Simport tubes with ball bearings in a Geno Grinder (for 10 min at 1000 rpm). The weighed samples were put into tin capsules (6 mm × 4 mm) and placed in a Costar 96-well plate for analysis and to determine δ^13^C values the samples were run in isotope ratio mass spectrometer (IRMS) [103].

### 4.3. Data Analysis

To determine and explain the variation in the each species, data were analyzed at two different levels. Firstly, all data used to treat all the observations simultaneously and secondly analyzed data at the species level for each functional group (such as herb, shrub and subshrub). Moreover, the differences in C, N, P, K, δ^13^C and LWP among functional groups were explored by One-way ANOVA. Principal component analysis (PCA) was conducted to assess the correlation among six traits in plant leaf. The general linear model (GLM) was used to compute the contribution of soil and plant group/family to the total variance of the leaf traits.

The phylogenetic tree for all the 37 plant species was constructed using Angiosperm Phylogeny Group III (APG III) classification of angiosperms [104], by the online tool Phylomatic (http://www.phylodiversity.net/phylomatic/phylomatic.html). The intensity of phylogenetic signals of all leaf traits was measured by using K statistics for leaf trait identification and confirmation regarding phylogenetically conservancy [105]. Phylogenetic independent contrasts (PIC) method was applied to eliminate phylogenetic error of correlations [106]. The “picante” package was used in R for phylogenetic analysis [107]. Then, Pearson correlation and PIC correlation coefficients were determined in the R package by using the “lm” function. Mean values of the leaf C, N, P, K, δ^13^C and LWP were mapped on the phylogenetic family tree for the identification of their phylogenetic patterns. Linear regressions were constructed to evaluate the bivariate relationship between the leaf traits of all plant species and plant life forms (such as herb, shrub and subshrub). All statistical analyses were performed by using R software (version 3.6.0, R Development Core Team 2018).

## 5. Conclusions

It is inferred that different leaf traits (C, N, P, LWP and δ^13^C, except K) showed convergent patterns for all plant life forms. The plants of GCG have evolved themselves, adapted and developed different mechanisms for their survival in the arid environment. The differences in the concentration pattern of leaf traits indicate the difference in their functional groups. Therefore, the plants in this study exhibited their special intrinsic features. Moreover, the leaf traits of desert plants in the GCG morpho-physiologically converged and the convergence pattern was not phylogenetic-specific. All the leaf traits were found phylogenetically conserved. Furthermore, marks of convergence found in the leaf traits of the GCG were most likely due to the environmental factors. The study might be helpful to understand the convergent adaptations/patterns of desert plants in the arid regions.

## Figures and Tables

**Table 1:** Species compositional list of 37 collected plants in different functional groups in the Guazhou Common Garden.

Species Name	Code	Family	Life Form	Phylogeny	Photosynthetic Pathway
*Phragmites australis*	Pa.	Poaceae	Herb	Monocots	C_3_
*Allium mongolicum*	Am.	Amaryllidaceae	Herb	Monocots	C_3_
*Karelinia caspia*	Kc.	Asteraceae	Herb	Dicots	C_3_
*Scorzonera pseudodivaricata*	Sp.	Asteraceae	Herb	Dicots	C_3_
*Halogeton glomeratus*	Hg.	Amaranthaceae	Herb	Dicots	C_4_
*Asterothamnus centraliasiaticus*	Ac.	Asteraceae	Herb	Dicots	C_3_
*Artemisia desertorum*	Ad.	Asteraceae	Herb	Dicots	C_3_
*Hedysarum scoparium*	Hs.	Fabaceae	Herb	Dicots	C_3_
*Sonchus oleraceus*	So.	Asteraceae	Herb	Dicots	C_3_
*Salsola ikoikovii*	Si.	Amaranthaceae	Herb	Dicots	C_4_
*Hedysarum multijugum*	Hm.	Fabaceae	Herb	Dicots	C_3_
*Zygophyllum gobicum*	Zg.	Zygophyllaceae	Herb	Dicots	C_3_
*Limonium aureum*	La.	Plumbaginaceae	Herb	Dicots	C_3_
*Lycium ruthenicum*	Lr.	Solanaceae	Shrub	Dicots	C_3_
*Tamarix chinensis*	Tc.	Tamaricaceae	Shrub	Dicots	C_3_
*Elaeagnus angustifolia*	Ea.	Elaeagnaceae	Shrub	Dicots	C_3_
*Calligonum mongolicum*	Cm.	Polygonaceae	Shrub	Dicots	C_4_
*Artemisia frigida*	Af.	Asteraceae	Shrub	Dicots	C_3_
*Gymnocarpos przewalskii*	Gp.	Caryophyllaceae	Shrub	Dicots	C_3_
*Sarcozygium xanthoxylon*	Sx.	Zygophyllaceae	Shrub	Dicots	C_3_
*Xanthoceras sorbifolia*	Xs.	Sapindaceae	Shrub	Dicots	C_3_
*Salsola arbuscula*	Sa.	Amaranthaceae	Shrub	Dicots	C_4_
*Caragana roborovskyi*	Cr.	Fabaceae	Shrub	Dicots	C_3_
*Caragana korshinskii*	Ck.	Fabaceae	Shrub	Dicots	C_3_
*Reaumuria songarica*	Rs.	Tamaricaceae	Shrub	Dicots	C_3_
*Ammopiptanthus mongolicus*	Amo.	Fabaceae	Shrub	Dicots	C_3_
*Nitraria sphaerocarpa*	Ns.	Nitrariaceae	Shrub	Dicots	C_3_
*Caryopteris mongholia*	Cam.	Lamiaceae	Subshrub	Dicots	C_3_
*Alhagi sparsifolia*	As.	Fabaceae	Subshrub	Dicots	C_3_
*Clematis tangutica*	Ct.	Ranunculaceae	Subshrub	Dicots	C_3_
*Bassia dasyphylla*	Bd.	Amaranthaceae	Subshrub	Dicots	C_3_
*Cynanchum chinense*	Cc.	Apocynaceae	Subshrub	Dicots	C_3_
*Sympegma regelii*	Sr.	Amaranthaceae	Subshrub	Dicots	C_3_
*Ephedra przewalskii*	Ep.	Ephedraceae	Subshrub	Gymnosperm	C_3_
*Sophora alopecuroides*	Sa.	Fabaceae	Subshrub	Dicots	C_3_
*Ephedra sinica*	Es.	Ephedraceae	Subshrub	Gymnosperm	C_3_
*Haloxylon ammodendron*	Ha.	Amaranthaceae	Subshrub	Dicots	C_4_

**Table 2:** Concentrations of analyzed leaf traits: as leaf carbon (C) concentration, leaf nitrogen (N) concentration, leaf phosphorus (P) concentration, leaf potassium (K) concentration, carbon isotope C^13^ (δ^13^C) concentration and water potential (ψ_w_) for plants three different life forms (such as herb, shrub, subshrub).

Life Form	Statistic	Leaf Traits					
		C (mg g^−1^)	N (mg g^−1^)	P (mg g^−1^)	K (mg g^−1^)	δ^13^C	LWP (MPa)
Herb	Mean	465.47	22.00	0.85	8.38	−24.88	−8.66
(*n* = 13)	G. Mean	454.53	20.75	0.78	7.05	-	-
	Max	772.99	40.38	1.97	12.12	−13.63	−3.01
	Min	298.86	12.99	0.43	4.32	−28.98	−19.60
	SE	30.94	2.23	0.11	0.65	1.40	1.24
	CV	0.24	0.37	0.46	0.28	−0.20	−0.52
Shrub	Mean	478.97	27.20	0.78	14.91	−24.47	−7.29
(*n* = 14)	G. Mean	467.52	25.57	0.74	14.06	-	-
	Max	639.65	53.40	1.29	26.35	−14.76	−4.24
	Min	262.25	13.52	0.42	8.08	−27.59	−16.33
	SE	27.75	2.73	0.07	1.47	1.05	1.00
	CV	0.22	0.38	0.35	0.37	−0.16	−0.51
Subshrub	Mean	441.01	26.84	0.95	11.16	−24.63	−6.96
(*n* = 10)	G. Mean	432.29	25.53	0.83	10.03	-	-
	Max	541.11	38.79	2.51	22.08	−13.62	−3.05
	Min	282.72	13.54	0.51	4.13	−28.13	−11.00
	SE	27.61	2.66	0.20	1.70	1.31	0.95
	CV	0.20	0.31	0.67	0.48	−0.17	−0.43
All	Mean	463.97	25.27	0.85	11.60	−24.66	−7.68
(*n* = 37)	SE	9.83	0.87	0.04	0.50	0.41	0.37
ANOVA Results							
Life form	F	0.40	1.32	0.45	6.87	0.03	0.67
	*P*	0.67	0.28	0.64	0.00	0.97	0.52

G. Mean = geometric mean; SE = standard error; CV = coefficient of variation; *n* = sample size; *p*-values are in bold when *p* < 0.05.

**Table 3:** General information about soil parameters in the Guazhou Common Garden.

Statistic	Soil Parameters						
	SOC (mg g^−1^)	STN (mg g^−1^)	STP (mg g^−1^)	SAP (mg g^−1^)	SWC	pH	SEC (µS/cm)
Mean	2.58	0.239	0.343	5.24	0.456	8.97	485.29
SE	1.49	0.138	0.198	3.02	0.263	5.18	280.19
CV	0.26	0.305	0.053	0.58	0.580	0.03	0.57

SE = standard error; CV = coefficient of variation; SOC = soil organic carbon; STN = soil total nitrogen; STP = soil total phosphorus; SAP = soil available phosphorus; SWC = soil water contents; SEC = soil electrical conductivity.

**Table 4:** Concentrations of analyzed leaf traits: as leaf carbon (C) concentration, leaf nitrogen (N) concentration, leaf phosphorus (P) concentration, leaf potassium (K) concentration, carbon isotope C^13^ concentration (δ^13^C) and leaf water potential (LWP) for C_3_, C_4_, legumes and nonlegumes species.

Plant Category	Statistic	Leaf Traits					
		C (mg g^−1^)	N (mg g^−1^)	P (mg g^−1^)	K (mg g^−1^)	δ^13^C	LWP (MPa)
C_3_	Mean	481.92	26.59	0.86	11.63	−26.25	−7.45
(*n* = 32)	SE	16.60	1.60	0.08	0.96	0.24	0.60
	CV	0.19	0.34	0.53	0.47	−0.05	−0.46
C_4_	Mean	349.08	16.87	0.81	11.45	−14.48	−9.14
(*n* = 5)	SE	29.21	1.49	0.09	2.06	0.52	2.70
	CV	0.19	0.20	0.24	0.40	−0.08	−0.66
ANOVA Results							
	F	9.20	5.53	0.05	0.00	336.07	0.85
	*P*	0.00	0.02	0.82	0.95	0.00	0.36
Legumes	Mean	488.89	27.00	0.61	12.61	−25.83	−6.79
(*n* = 7)	SE	26.40	2.09	0.04	2.55	0.57	1.42
	CV	14.29	20.45	17.66	53.56	−5.88	−55.13
Nonlegumes	Mean	458.15	24.87	0.91	11.37	−24.38	−7.88
(*n* = 30)	SE	19.53	1.79	0.08	0.91	0.85	0.70
	CV	23.35	39.40	50.12	43.96	−19.20	−48.84
ANOVA Results							
	F	0.52	0.30	2.91	0.31	0.64	0.46
	*P*	0.48	0.58	0.10	0.58	0.43	0.50

SE = standard error; CV = coefficient of variation; *n* = sample size; *p*-values are in bold when *p* < 0.05.

**Table 5:** Phylogenetically independent contrast (PIC) correlations (above the diagonal) and Pearson’s correlation (below the diagonal) for the different leaf traits at the species level.

	C	N	P	K	δ^13^C	LWP
C		0.350 *	0.341 *	0.033	−0.099	−0.180
N	0.333 *		0.709 **	−0.048	−0.417 *	0.059
P	0.201	0.586 **		−0.494 **	−0.332 *	−0.409 *
K	−0.069	0.023	−0.154		−0.024	0.610 **
δ^13^C	−0.404 *	−0.294	0.004	0.038		−0.135
LWP	−0.243	0.052	−0.122	0.258	–0.162	

Correlation is significant at ** *p* ≤ 0.01 and * *p* ≤ 0.05.

**Table 6:** General linear model (GLM) summary for the effects of taxonomy and soil factors on the concentrations of leaf traits.

Leaf Traits	Total Effects (*r*^2^, %)		
	Full	Taxonomy	Soil
C	65.61	57.76	7.84
N	82.02	73.65	8.37
P	90.38	82.18	8.19
K	39.32	35.44	3.88
δ^13^C	94.87	76.45	18.42
LWP	31.14	30.84	0.30

Soil factors: pH, EC, SWC in 0–20 cm.

## Supplementary Materials

The following are available online at https://www.mdpi.com/2223-7747/9/8/990/s1, Table S1: Phylogenetic signals of the different leaf traits. Table S2: The loading of six leaf traits on the three principal components analysis (PCA) axes at the species level (*N* = 37). Table S3: Correlation matrix for macro-elements of plant and soil., Table S4: Correlation matrix for soil elements and soil variables. Table S5: Concentrations (Mean ± SE) of analyzed leaf traits for 37 plant species: C, carbon (mg g^−1^); N, nitrogen (mg g^−1^); P, phosphorus (mg g^−1^); K, potassium (mg g^−1^); δ^13^C, carbon isotope ^13^C; and LWP, water potential in MPa (ψ_w_). The differences among species were assessed by one-way analysis of variance (ANOVA). Table S6: Concentrations (Mean ± SE) of analyzed leaf traits for 18 families: C, carbon (mg g^−1^); N, nitrogen (mg g^−1^); P, phosphorus (mg g^−1^); K, potassium (mg g^−1^); δ^13^C, carbon isotope ^13^C; and LWP, water potential in MPa (ψ_w_). The differences among families were assessed by one-way analysis of variance (ANOVA). Figure S1: Phylogenetic distributions patterns of leaf traits: leaf carbon concentration (C); leaf nitrogen concentration (N); leaf phosphorus concentration (P); leaf potassium concentration (K); carbon isotope concentration (δ^13^C); and leaf water potential (Ψ_l_) concentrations (mean ± SE) at the species level. Figure S2: Phylogenetic distributions patterns of leaf traits: leaf carbon concentration (C); leaf nitrogen concentration (N); leaf phosphorus concentration (P); leaf potassium concentration (K); carbon isotope concentration (δ^13^C); and leaf water potential (Ψ_l_) concentrations (mean ± SE) at the family level.
