# Supplementary material for: Convergent Variations in the Leaf Traits of Desert Plants
Source: Plants (Basel). 2020 Aug 4;9(8):990. doi: 10.3390/plants9080990 (PMC7463800; doi:10.3390/plants9080990)
Supplement: Supplementary file 1 [file plants-09-00990-s001.pdf]

Convergent Variations in the Leaf traits of Desert Plants

Muhammad Adnan Akram [1], Xiaoting Wang [1], Weigang Hu [1], Junlan Xiong [1], Yahui Zhang [1], Yan Deng [1], Jinzhi Ran [1]* and Jianming Deng [1]*

State Key Laboratory of Grassland Agro-Ecosystem, School of Life Sciences, Lanzhou University, Lanzhou, Gansu 730000, China
* Corresponding author: ranjz@lzu.edu.cn; dengjm@lzu.edu.cn

Supplementary information

Table S1 Phylogenetic signals of the different leaf traits.

Table S2 The loading of six leaf traits on the three principal components analysis (PCA) axes at the species level (N=37).

Table S3 Correlation matrix for macro-elements of plant and soil.

Table S4 Correlation matrix for soil elements and soil variables.

Table S5 Concentrations (Mean ± SE) of analyzed leaf traits for 37 plant species: as C, carbon (mg g^{-1}); N, nitrogen (mg g^{-1}); P, phosphorus (mg g^{-1}); K, potassium (mg g^{-1}); $\delta^{13}C$, carbon isotope ^{13}C and LWP, water potential in MPa (ψw). The differences among species were assessed by one-way analysis of variance (ANOVA).

Table S6 Concentrations (Mean ± SE) of analyzed leaf traits for 18 families: as C, carbon (mg g^{-1}); N, nitrogen (mg g^{-1}); P, phosphorus (mg g^{-1}); K, potassium (mg g^{-1}); $\delta^{13}C$, carbon isotope ^{13}C and LWP, water potential in MPa (ψw). The differences among families were assessed by one-way analysis of variance (ANOVA).

Figure S1 Phylogenetic distributions patterns of leaf traits: leaf carbon concentration (C); leaf nitrogen concentration (N); leaf phosphorus concentration (P); leaf potassium concentration (K); carbon isotope concentration (C^{13}) and leaf water potential (Ψ_l) concentrations (mean ± SE) at the species level.

Figure S2 Phylogenetic distributions patterns of leaf traits: leaf carbon concentration (C); leaf nitrogen concentration (N); leaf phosphorus concentration (P); leaf potassium concentration (K); carbon isotope concentration ($\delta^{13}C$) and leaf water potential (Ψ_l) concentrations (mean ± SE) at the family level.

Table S1 Phylogenetic signals of the different leaf traits.

**Traits**	***K -value***	***P -value***
**C**	0.348	0.010**
**N**	0.282	0.050*
**P**	0.173	0.515
**K**	0.123	0.732
**$\delta^{13}C$**	0.283	0.155
**LWP**	0.114	0.682

** $P \leq 0.01$and * $P \leq 0.05$ represent significant phylogenetic signal in the corresponding traits

Table S2 The loading of 6 leaf traits on the three principal components analysis (PCA) axes at the species level (*N*=37).

**Variables**	**PC1**	**PC2**	**PC3**
C	0.717	-0.041	-0.520
N	0.810	0.190	0.358
P	0.594	0.604	0.342
K	-0.202	0.810	0.063
$\delta^{13}C$	-0.624	0.558	0.110
LWP	-0.052	-0.545	0.748
Total variation explained	32.7%	22.5%	18.5%

Table S3 Correlation matrix for macro-elements of plant and soil

	**SOC**	**STN**	**STP**
**C**	-0.072	-0.104	-0.285
**N**	-0.009	0.030	-0.091
**P**	0.510**	0.453**	0.191

SOC= soil organic carbon, STN= soil total nitrogen, STP= soil total phosphorus. Correlation is significant at ** $P \leq 0.01$and * $P \leq 0.05$

Table S4 Correlation matrix for soil elements and soil variables

	SOC	STN	NH4	NO3	STP	SAP	SMC	pH	SEC
**SOC**	1								
**STN**	0.932**	1							
**NH4**	0.460*	0.534*	1						
**NO3**	-0.102	-0.018	0.272	1					
**STP**	0.593**	0.573**	0.289	0.042	1				
**SAP**	0.145	0.226	-0.177	-0.096	0.305	1			
**SMC**	0.514*	0.566**	0.649**	0.428	0.203	-0.379	1		
**pH**	-0.306	-0.346	-0.296	-0.294	0.044	0.263	-0.567**	1	
**SEC**	0.014	0.165	0.243	0.279	-0.232	-0.052	0.399	-0.641**	1

Correlation is significant at ** $P \leq 0.01$and * $P \leq 0.05$

Table S5 Concentrations (Mean ± SE) of analyzed leaf traitsfor37 plant species: as C, carbon (mg g^{-1}); N, nitrogen (mg g^{-1}); P, phosphorus (mg g^{-1}); K, potassium (mg g^{-1}); $\delta^{13}C$, carbon isotope ^{13}C and LWP, water potential in MPa (ψw). The differences among species were assessed by one-way analysis of variance (ANOVA).

Plant Name	Statistic	C	N	P	K	$\delta^{13}C$	LWP
Allium mongolicum	Mean	470.74	40.38	1.97	7.25	-24.38	-9.18
	SE	19.45	0.77	0.04	0.12	0.39	1.15
Artemisia desertorum	Mean	772.99	29.88	1.02	12.12	-25.78	-13.36
	SE	24.36	0.61	0.02	0.10	0.75	0.20
Asterothamnus centraliasiaticus	Mean	449.05	27.89	0.88	9.07	-26.80	-7.60
	SE	27.58	0.98	0.02	0.62	0.41	0.15
Halogeton glomeratus	Mean	298.86	16.97	0.91	8.04	-13.98	-8.13
	SE	18.62	1.33	0.01	0.23	0.36	0.16
Hedysarum multijugum	Mean	405.04	24.94	0.66	10.80	-27.51	-3.91
	SE	23.14	0.82	0.03	0.25	1.26	0.04
Hedysarum scoparium	Mean	473.07	19.56	0.63	9.48	-26.86	-3.01
	SE	18.67	0.99	0.05	0.31	0.12	0.87
Karelinia caspia	Mean	448.91	28.14	0.81	4.32	-27.79	-7.81
	SE	7.01	0.43	0.07	0.37	0.81	0.18
Limonium aureum	Mean	455.40	13.33	0.50	7.95	-27.47	-11.83
	SE	18.42	0.27	0.00	0.32	0.20	0.10
Phragmites australis	Mean	492.26	23.18	0.81	10.00	-25.39	-7.09
	SE	24.76	0.82	0.04	0.42	0.74	0.04
Salsola ikoikovii	Mean	338.96	15.54	0.87	6.14	-13.63	-19.60
	SE	19.28	0.31	0.08	0.09	0.20	1.07
Scorzonera pseudodivaricata	Mean	538.12	14.52	0.50	11.26	-26.92	-7.27
	SE	22.23	1.01	0.03	0.34	0.19	0.09
Sonchus oleraceus	Mean	467.76	18.66	1.01	6.95	-28.98	-3.67
	SE	19.73	1.06	0.02	0.34	0.20	0.05
Zygophyllum gobicum	Mean	439.96	12.99	0.43	5.61	-27.94	-10.06
	SE	22.58	1.82	0.07	0.27	0.66	0.17
Ammopiptanthus mongolicus	Mean	614.79	32.68	0.71	26.35	-25.22	-7.04
	SE	24.11	0.66	0.01	0.21	0.79	1.11
Artemisia frigida	Mean	639.65	33.79	1.21	16.79	-26.68	-4.39
	SE	24.47	0.69	0.08	0.35	1.28	0.03
Calligonum mongolicum	Mean	444.09	15.89	1.02	10.82	-14.76	-6.19
	SE	10.24	1.42	0.04	0.34	1.10	0.93
Caragana korshinskii	Mean	511.55	26.74	0.42	14.01	-23.65	-5.34
	SE	17.50	0.42	0.02	0.06	0.60	1.36
Caragana roborovskyi	Mean	501.08	25.74	0.72	13.66	-25.13	-12.81
	SE	21.48	1.11	0.04	0.19	0.43	1.08
Elaeagnus angustifolia	Mean	542.67	37.40	1.02	9.65	-25.83	-4.88
	SE	20.79	0.86	0.11	0.79	0.40	0.01
Gymnocarpos przewalskii	Mean	426.86	16.27	0.62	12.35	-24.57	-4.36
	SE	28.45	0.13	0.01	0.27	0.13	0.22
Lycium ruthenicum	Mean	379.19	30.04	0.76	8.08	-27.14	-4.24
	SE	11.22	1.46	0.05	0.24	0.12	0.05
Nitraria sphaerocarpa	Mean	449.05	53.40	1.29	11.90	-26.38	-8.29
	SE	11.34	1.84	0.06	0.43	0.57	0.91
Reaumuria songarica	Mean	262.25	25.51	0.47	25.00	-27.59	-5.09
	SE	12.80	0.56	0.03	0.04	0.24	0.04
Salsola arbuscula	Mean	380.79	22.41	0.73	15.45	-16.39	-7.40
	SE	14.43	0.55	0.12	0.44	0.07	0.09

Continued…..

**Plant Name**	**Statistic**	**C**	**N**	**P**	**K**	**δ^{13}C**	**LWP**
Sarcozygium xanthoxylon	Mean	432.99	13.52	0.43	17.32	-27.08	-4.33
	SE	23.27	2.44	0.05	0.36	0.11	0.14
Tamarix chinensis	Mean	518.40	22.96	0.70	18.12	-26.26	-11.33
	SE	9.41	0.77	0.06	1.13	0.28	0.12
Xanthoceras sorbifolia	Mean	602.50	24.46	0.83	9.26	-25.88	-16.33
	SE	11.12	0.52	0.10	0.46	0.89	0.82
Alhagi sparsifolia	Mean	415.83	23.55	0.60	8.64	-27.65	-11.00
	SE	4.34	0.50	0.02	0.24	0.68	0.05
Bassia dasyphylla	Mean	326.53	19.43	0.62	22.08	-24.70	-5.26
	SE	25.70	1.37	0.11	0.61	0.18	0.80
Caryopteris mongholia	Mean	541.11	38.79	2.51	9.94	-26.88	-11.00
	SE	11.55	0.91	0.03	0.48	0.08	0.08
Clematis tangutica	Mean	487.61	24.12	0.78	11.32	-26.76	-5.79
	SE	15.64	0.67	0.04	0.38	0.50	0.18
Cynanchum chinense	Mean	466.11	30.81	0.81	9.02	-28.13	-5.76
	SE	27.62	0.92	0.04	0.08	0.06	0.65
Ephedra przewalskii	Mean	509.09	17.54	0.72	4.13	-25.19	-9.47
	SE	9.50	1.04	0.05	0.21	0.69	0.19
Ephedra sinica	Mean	506.42	33.14	0.83	10.01	-24.81	-9.59
	SE	15.24	0.22	0.07	0.37	0.56	0.16
Haloxylon ammodendron	Mean	282.72	13.53	0.51	16.82	-13.62	-4.18
	SE	2.12	0.14	0.05	0.33	0.13	0.11
Sophora alopecuroides	Mean	500.86	35.81	0.52	5.33	-24.82	-4.45
	SE	17.89	0.40	0.05	0.25	0.72	0.95
Sympegma regelii	Mean	373.85	31.64	1.62	14.35	-23.77	-3.05
	SE	17.03	0.69	0.12	0.20	0.15	0.02
**Mean (37-species)**	Mean	463.98	25.27	0.85	11.60	-24.66	-7.68
	SE	9.83	0.87	0.04	0.50	0.41	0.37
	F-value	28.63	85.68	52.70	185.00	56.63	43.91
	P-value	**<0.001**	**<0.001**	**<0.001**	**<0.001**	**<0.001**	**<0.001**

SE = standard error; *P*-values are in bold when $P < 0.001$.

Table S6 Concentrations (Mean ± SE) of analyzed leaf traitsfor18 families: as C, carbon (mg g^{-1}); N, nitrogen (mg g^{-1}); P, phosphorus (mg g^{-1}); K, potassium (mg g^{-1}); δ13C, carbon isotope ^{13}C and LWP, water potential in MPa (ψw). The differences among families were assessed by one-way analysis of variance (ANOVA).

Family Name	Statistic	C	N	P	K	$\delta^{13}C$	LWP
Asteraceae	Mean	552.75	25.48	0.91	10.08	-27.16	-7.35
	SE	1.11	0.51	0.03	0.10	0.14	0.04
Lamiaceae	Mean	541.11	38.79	2.51	9.94	-26.88	-11.00
	SE	11.55	0.91	0.03	0.48	0.08	0.08
Solanaceae	Mean	379.19	30.04	0.76	8.08	-27.14	-4.24
	SE	11.22	1.46	0.06	0.24	0.12	0.05
Apocynaceae	Mean	466.11	30.81	0.81	9.02	-28.13	-5.76
	SE	27.62	0.92	0.07	0.08	0.06	0.65
Amaranthaceae	Mean	333.62	19.93	0.88	13.82	-17.68	-7.94
	SE	7.74	0.68	0.03	0.11	0.07	0.26
Caryophyllaceae	Mean	426.86	16.27	0.62	12.35	-24.57	-4.36
	SE	28.45	0.13	0.01	0.27	0.13	0.22
Polygonaceae	Mean	444.09	15.89	1.02	10.82	-14.76	-6.19
	SE	10.24	1.42	0.04	0.34	1.10	0.93
Plumbaginaceae	Mean	455.40	13.33	0.50	7.95	-27.47	-11.83
	SE	18.42	0.27	0.03	0.32	0.20	0.10
Tamaricaceae	Mean	390.32	24.23	0.59	21.56	-26.93	-8.21
	SE	7.28	0.39	0.04	0.55	0.05	0.06
Zygophyllaceae	Mean	436.48	13.25	0.43	11.47	-27.51	-7.20
	SE	19.72	1.67	0.04	0.30	0.29	0.12
Fabaceae	Mean	488.89	27.00	0.61	12.61	-25.83	-6.79
	SE	8.01	0.11	0.01	0.12	0.28	0.17
Elaeagnaceae	Mean	542.67	37.40	1.02	9.65	-25.83	-4.88
	SE	20.79	0.86	0.02	0.79	0.40	0.01
Sapindaceae	Mean	602.50	24.46	0.83	9.26	-25.88	-16.33
	SE	11.12	0.52	0.10	0.46	0.89	0.82
Nitrariaceae	Mean	449.05	53.40	1.29	11.90	-26.38	-8.29
	SE	11.34	1.84	0.06	0.43	0.57	0.91
Ranunculaceae	Mean	487.61	24.12	0.78	11.32	-26.76	-5.79
	SE	15.64	0.67	0.04	0.38	0.50	0.18
Amaryllidaceae	Mean	470.74	40.38	1.97	7.25	-24.38	-9.18
	SE	19.45	0.77	0.04	0.12	0.39	1.15
Poaceae	Mean	492.26	23.18	0.81	10.00	-25.39	-7.09
	SE	24.76	0.82	0.04	0.42	0.74	0.04
Ephedraceae	Mean	507.76	25.34	0.77	7.07	-25.01	-9.53
	SE	4.79	0.44	0.04	0.25	0.34	0.08
**Mean (18-families)**	Mean	470.41	26.85	0.95	10.79	-25.20	-7.89
	SE	9.41	1.41	0.07	0.44	0.47	0.41
	F-value	16.56	123.47	128.10	80.22	56.40	36.58
	P-value	**<0.001**	**<0.001**	**<0.001**	**<0.001**	**<0.001**	**<0.001**

SE = standard error; *P*-values are in bold when $P< 0.001$.

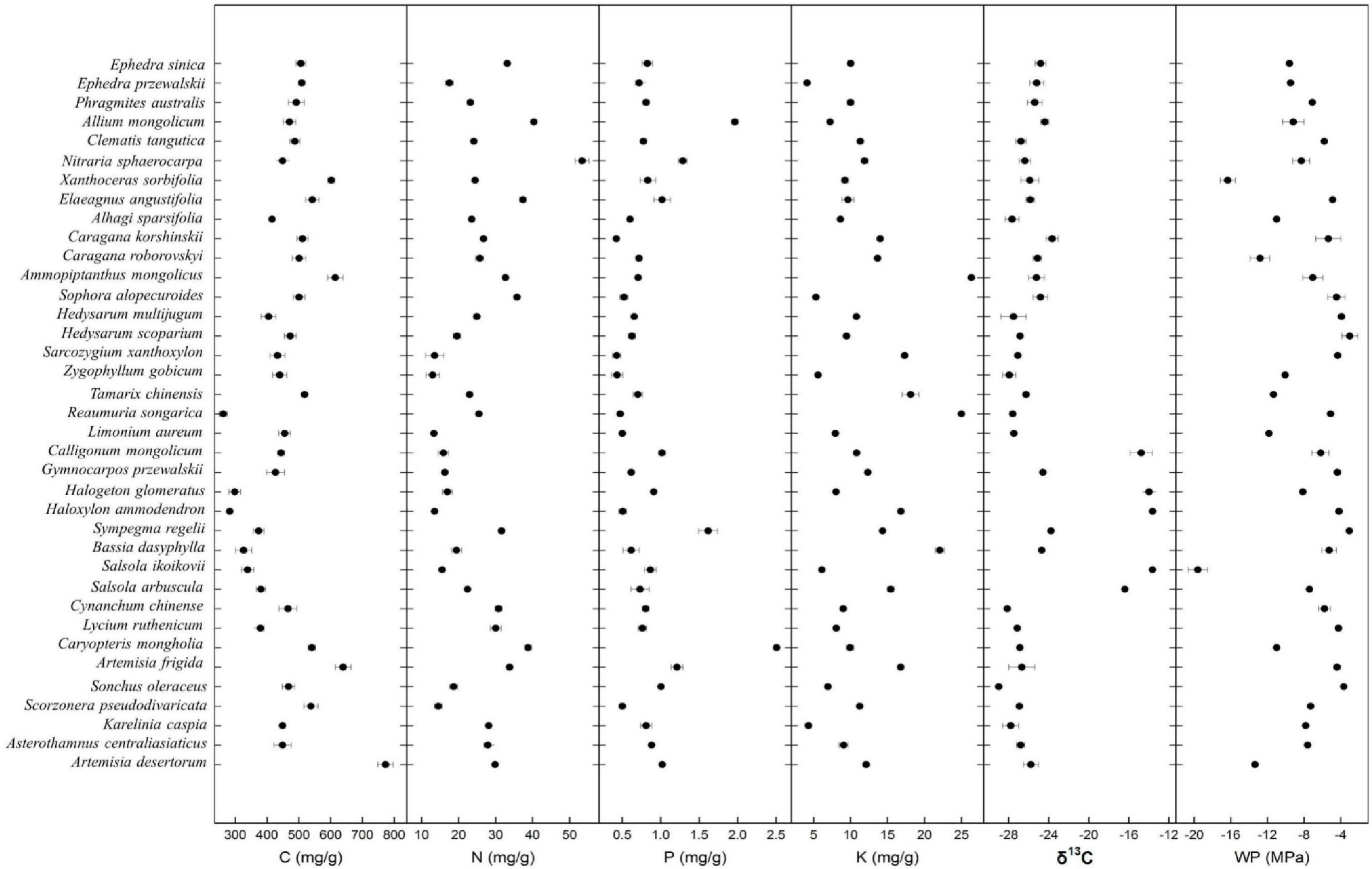

Fig.S1 Phylogenetic distributions patterns of leaf traits: leaf carbon concentration (C); leaf nitrogen concentration (N); leaf phosphorus concentration (P); leaf potassium concentration (K); carbon isotope concentration ($\delta^{13}C$) and leaf water potential (Ψ_l) concentrations (mean ± SE) at the species level.

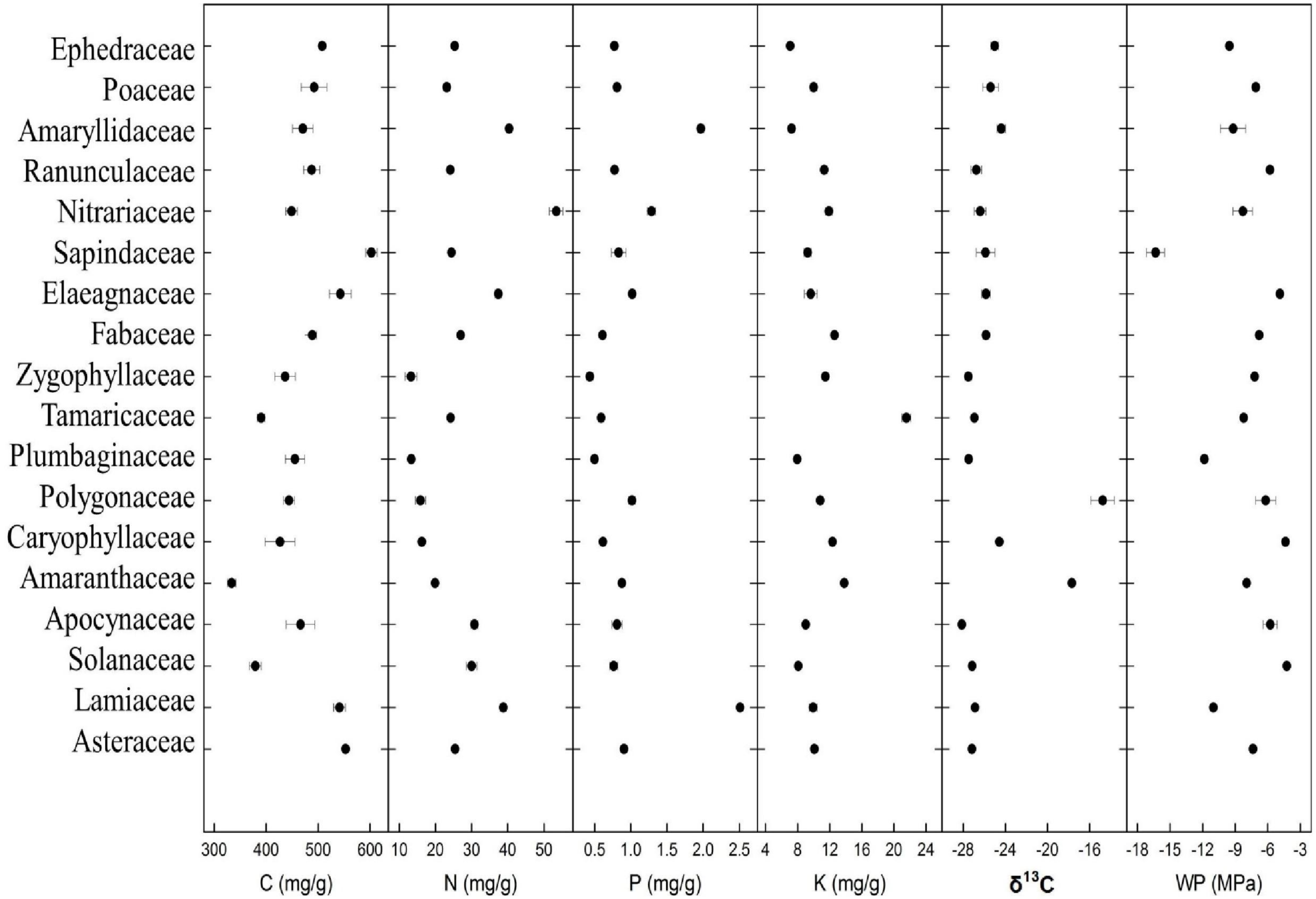

Fig.S2 Phylogenetic distributions patterns of leaf traits: leaf carbon concentration (C); leaf nitrogen concentration (N); leaf phosphorus concentration (P); leaf potassium concentration (K); carbon isotope concentration ($\delta^{13}C$) and leaf water potential (Ψ_l) concentrations (mean ± SE) at family level.
